# Early Steroid-Induced Osteonecrosis of Rabbit Femoral Head and *Panax notoginseng* Saponins: Mechanism and Protective Effects

**DOI:** 10.1155/2015/719370

**Published:** 2015-03-16

**Authors:** Hui Qiang, Huitong Liu, Ming Ling, Kunzheng Wang, Chen Zhang

**Affiliations:** ^1^The Second Department of Orthopaedics, Shaanxi Provincial People's Hospital, The Third Affiliated Hospital of Xi'an Jiaotong University, Xi'an, Shaanxi 710068, China; ^2^The First Department of Orthopaedics, Fuzhou Second Hospital of Xiamen University, Fuzhou, Fujian 350007, China; ^3^The First Department of Orthopaedics, The Second Affiliated Hospital of Xi'an Jiaotong University, Xi'an, Shaanxi 710004, China

## Abstract

*Background.* This study was aimed at investigating the pathogenesis of oxidative stress in steroid-induced avascular necrosis of the femoral head (SANFH) and at exploring the mechanism and protective effects of *Panax notoginseng* saponins (PNS) on early SANFH. *Methods.* 80 adult New Zealand rabbits were randomly divided into control group, model group, and PNS group. In model group, equine serum was injected into auricular vein; then methylprednisolone was injected into gluteus. In PNS group, PNS was applied for 14 consecutive days before methylprednisolone management. At different time points, serum and femoral heads were prepared for T-AOC, SOD, GSH-PX, ·OH, and MDA determination. Two weeks after steroid management, all femoral heads were assessed with MRI and HE staining. *Results.* Typical early osteonecrosis symptoms were observed in model group. Our results showed that PNS could significantly ameliorate the decrease of T-AOC level, improve SOD and GSH-PX activity, suppress ·OH ability, and augment MDA level. Besides, PNS improved MRI and pathological changes of the femoral head, markedly reducing the incidence of osteonecrosis. *Conclusion.* Based on our research, we found oxidative stress played a positive role in the occurrence of SANFH where reactive oxygen species was the direct cause. PNS could protect rabbits against early steroid-induced osteonecrosis of femoral head by its antioxidative effect.

## 1. Introduction

Steroid-induced avascular necrosis of the femoral head (SANFH) has been one of the hard nuts for orthopedists. Researchers home and abroad have so far proposed few hypotheses for the pathogenesis of this disease, including lipid metabolism disorder, intravascular coagulation, microvascular injury, and intraosseous hypertension [1–4]. However, the exact mechanism of SANFH still remains unclear.

*Panax notoginseng* saponins (PNS) is an active ingredient extracted from Chinese herbal medicine* Panax notoginseng* [5]. As it has advantages of stable structure, easy access, and less toxic effects, it has been widely used in clinical practice. In particular, its antioxidant effect has drawn much attention. In our previous researches [6], we have confirmed that PNS has protective effect on bone marrow mesenchymal stem cells (BMSCs) cultured* in vitro*. However, there exist differences among cells cultured in animal bone tissues and* in vitro*. Organisms have more complex life activities and influencing factors and each tissue, including bone tissue, functions in a complex but balanced internal environment. On the other hand, bone tissues, as an organ with complete physiological function constituted by various types of cells, are different from a holistic organism. Although* in vitro* cell experiments can expound the antioxidant role of PNS, they fail to prove the effect of PNS on the whole body.

In this study, we established early SANFH rabbit models by equine serum combined with steroid and observed the systematic (serum) and local (femoral head) changes of oxidative stress and antioxidative systems in the pathogenesis of SANFH and the regulative effect of PNS on oxidative stress, hoping to explore the pathogenesis of SANFH, thus providing new theoretic evidence for the treatment and prevention of this disease.

## 2. Materials and Methods

### 2.1. Chemicals and Reagents

PNS injection (Batch number 08FL03, 250 mg/10 mL/ampoul) was purchased from Kunming Pharmaceutical Corporation (Kunming, China), and its purity was >99% by high-performance liquid chromatography. Methylprednisolone was purchased from Prifer Pharmaceutical Corporation, US. Equine serum was purchased from Nanjing Keygen Biotech Co. Ltd (Nanjing, China). Total antioxidative capability (T-AOC), superoxidedismutase (SOD), glutathione peroxidase (GSH-PX), hydroxyl radical (·OH) and malondialdehyde (MDA) assay kit were purchased from Nanjing Jiancheng Bioengineering Institute (Nanjing, China). Diazepam injection was purchased from Xudong Haipu Pharmaceutical Co., Ltd (Shanghai, China). And the ketamine injection was purchased from Gutian Pharma Co., Ltd (Fujian, China).

### 2.2. Ethical Statement

Rabbit care and use were conducted in strict accordance with the recommendations in the* Guide for the Care and Use of Laboratory Animals of the National Institutes of Health*. All protocols were approved by Medical Animal Studies Committee of Xi'an Jiaotong University, China. New Zealand rabbits were housed and bred at temperature-controlled room, received the standard chew diet, and were kept on a cycle of 12 h light and 12 h dark, with the darkness starting from 19:00. The rabbits were sacrificed by air embolism. All efforts were made to minimize suffering and the only procedures performed on the dead animals were getting blood and femoral head.

### 2.3. Animal Experiments and Grouping

A total of 80 healthy adult New Zealand rabbits (SPF, Certificate of Quality No. 2008-008) were purchased from the Animal Center of Xi'an Jiaotong University. Half of the animals was males and half was females, body weight 2.6–3.2 kg, averaging 2.9 kg. All animals could eat and drink freely at a room temperature of 25 ± 3°C. After 1 week, the rabbits were weighed accurately and randomly divided into 3 groups: normal control group (*n* = 20), model group (*n* = 30), and PNS group (*n* = 30). Rabbits in all the three groups received normal feeding. Rabbits in the PNS group were injected with PNS 50 mg/kg for 14 days before the first administration of methylprednisolone.

### 2.4. Establishment of SANFH Animal Models

 Rabbits were tied to the laboratory table, and the hair around the auricular injection site was removed to establish models according to method adopted by Wang et al. [7]. The auricular skin was sterilized for a second time with 70% medical alcohol. Blood flow was temporarily blocked at the proximal end of puncture point at auricular vein. When the vein dilated, the vein was punctured, and when blood was drawn out the block was stopped. After that, equine serum was slowly injected. Meanwhile, the respiration of rabbits should be observed to control the speed of injection (<1.0 mL/min). The first dose of equine serum was 10 mL/kg and, after 3 weeks, a second dose of 6 mL/kg was injected. After an interval of 2 weeks, 40 mg/kg methylprednisolone was injected intragluteally daily for 3 days.

### 2.5. MRI Examination

Fourteen days after steroid injection, MRI imaging was performed. When the rabbits were fastened, 3.5 mg/kg diazepam injection was injected into the deltoid. After 15 min, 25 mg/kg ketamine was injected. Then coronal scan of bilateral femoral heads of all rabbits was performed with Philips Gyroscan T5-NT MRI. The indexes were as follows: T1WI (TR 685 ms, TE 25 ms), T2WI (TR 1456 ms, TE 70 ms), Stir (TR 1456 ms, TE 70 ms), slice thickness 1.2 mm, interval 0.12 mm, and matrix (reconstruction 256 × matrix scan 256). The MRI results of early SANFH were then compared among the groups.

### 2.6. Sample Collection and Detection

#### 2.6.1. Blood Sample Collection and Detection

Blood samples (2 mL each time) were collected 1 day before and 3, 5, 7, and 14 days after steroid injection from auricular vein (after 12 hours of food-fast and 8 hours of water-fast). After the blood samples stood for a while, the supernatant was taken, which was then centrifuged for 10 min (4°C, 4000 r/min). After being isolated, the serum sample was placed in a microtube and preserved at −70°C. Then, the assay kit was adopted for T-AOC, SOD, GSH-PX, ·OH, and MDA detection.

#### 2.6.2. Femoral Head Sample Collection and Detection

On days 3, 5, 7, and 14 before and after steroid injection, femoral heads of 3 rabbits in the experiment groups were taken, washed with PBS, dried with sterile gauze, and placed in a −70°C refrigerator. After being weighted, they were quickly put into a mortar containing liquid nitrogen and were ground. Then, according to the tissue/water ratio of 1 : 9, ice-salt mixture was added to the ground bone, which was then put into an electric homogenizer. After that, the 10% homogenate was centrifuged for 10 min (4°C, 3000 r/min), and the supernatant was isolated and preserved at −70°C. The BCA assay kit was used to determine the protein content. The assay kit was adopted for T-AOC, SOD, GSH-PX, ·OH, and MDA detection.

### 2.7. Histological Examination

The femoral heads of rabbits in the experimental groups were taken 14 days after steroid injection for pathological observation. The procedures are illustrated below: after ketamine injection, the rabbits were tied onto the experimental table for skin preparation and sterilization. In a sterilized condition, the femoral heads were taken out. Firstly, the general features of the bones were observed. Secondly, the femoral heads were cut open along the coronal section and washed with PBS, which were then put into 10% formaldehyde solution for 3 days. Thirdly, the femoral heads were decalcified in 15% EDTA buffer solution. Fourthly, after complete decalcification, the bones were dehydrated gradually. After that, they were vitrificated by dimethylbenzene and embedded in paraffin. The paraffin block was cut into 5 *μ*m slices for HE staining. The signal acquisition and analysis system from Leica Cooperation (W550CW, Germany) was used for observation, analysis, and assessment.

### 2.8. Statistical Analysis

SPSS13.0 software (SPSS, US) was adopted for One-Way ANOVA and Chi-squared tests. Measurement data were presented as mean ± standard deviation ($\overset{-}{x}\pm s$). SNK test was employed for group comparison. The significance level *P* < 0.05 was supposed to be significant.

## 3. Results

### 3.1. MRI Results

**Figure 1:**
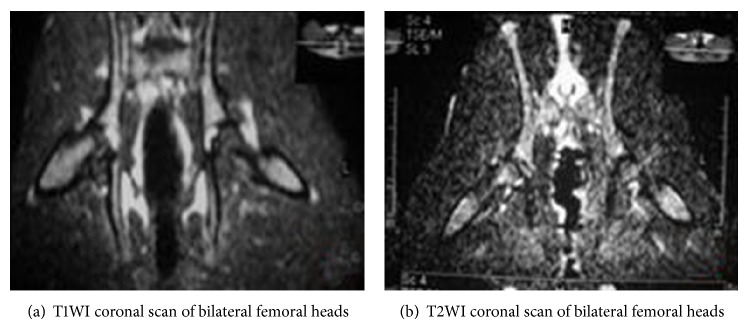
MRI imaging of bilateral femoral heads of rabbits in the control group.

**Figure 2:**
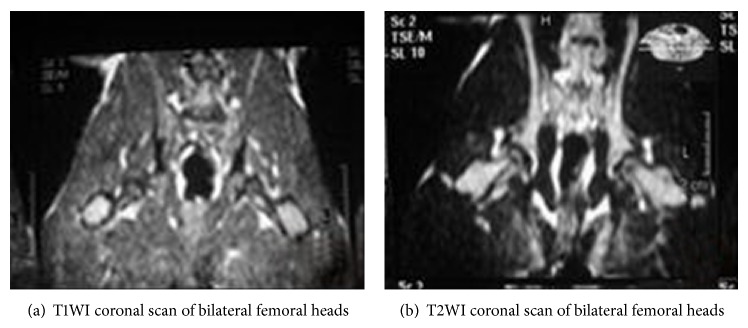
MRI imaging of bilateral femoral heads of rabbits in the model group.

**Figure 3:**
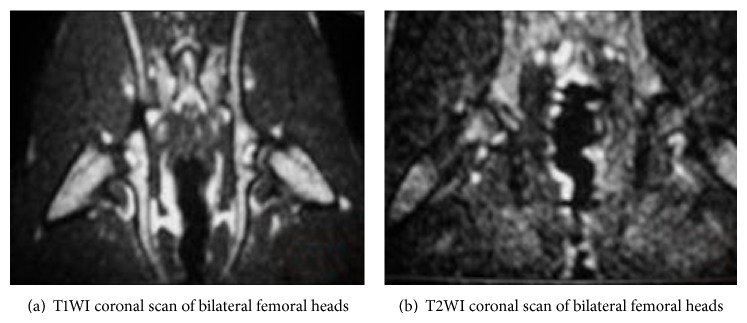
MRI imaging of bilateral femoral heads of rabbits in the PNS group.

The normal controls presented yellow bone marrow signal; T1W1 and T2W1 showed homogeneous and moderate-intensity signals (Figure 1). In the model group, bilateral femoral heads of most rabbits displayed abnormal MRI signals, showing lipid or myxoid changes. The intensity of T1WI signals was significantly reduced, showing line-like low-signal zone, while T2WI showed heterogeneous high-signal intensity (Figure 2). In the PNS group, T1WI signal intensity was stronger while T2WI signal intensity was weaker compared with that in the model group (Figure 3).

The MRI results indicated that there was no osteonecrosis in the control group, and the incidence of osteonecrosis in the model and PNS group was 1/13 (69.23%) and 3/15 (20.00%), respectively, suggesting significant difference between the two groups (*P* < 0.05).

### 3.2. Results of Histological Observations

#### 3.2.1. General Features

**Figure 4:**
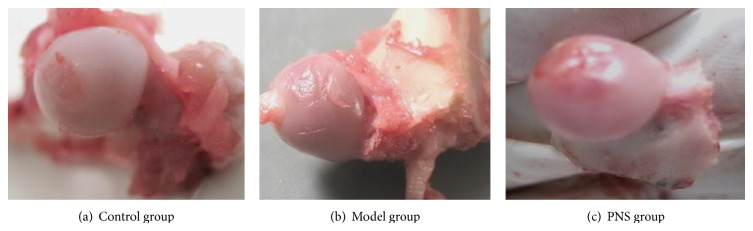
Outlook of rabbit femoral heads in the three groups.

The femoral heads of the controls looked normal. No collapse, hyperplasia of the synovial membrane of the articular capsule, or articular cavity effusion was observed. The articular cartilage surface was smooth and clean, with healthy color (Figure 4(a)). Two weeks after steroid injection, femoral heads had normal shape. No collapse, obvious hyperplasia of the synovial membrane of the articular capsule, or articular cavity effusion was observed. The articular cartilage surface was comparatively smooth, but the right upper quadrant of some bones showed a dark color and occasional petechiae (Figure 4(b)). In the PNS group, the bones looked normal, without apparent petechiae (Figure 4(c)).

#### 3.2.2. Results of HE Staining

**Figure 5:**
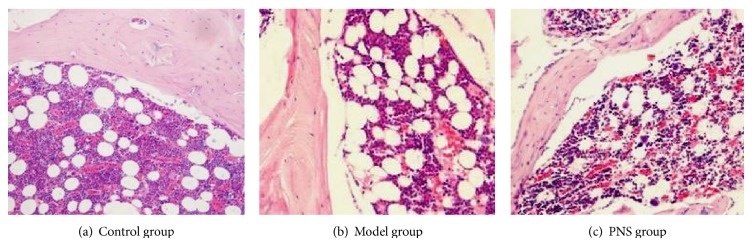
Results of HE staining of the femoral heads in the three groups (×200).

In the control group, there were rich hematopoietic cells and relatively fewer lipocytes. The bone trabeculas were regularly arrayed, with complete structure, clearly visible osteocytes and a few empty lacunaes (Figure 5(a)). In the model group, typical osteonecrosis was presented. The necrotic areas were located in the spongy bone near the neck of femur and subchondral area. Bone marrow structure disturbance, marrow cell necrosis, debris assembly, and bone marrow pimelosis were visible. The lipocytes were enlarged. Bone trabeculas turned thinner, and many empty lacunaes were observed. Margination of nucleus, flattening, poor staining, and karyopyknosis were visible (Figure 5(b)). In the PNS group, there were a few lipocytes, and no apparent necrotic debris was noted. The bone trabeculas regularly arrayed, and only a few empty lacunaes were observed (Figure 5(c)).

The histological examination showed that no osteonecrosis occurred in the control group; the incidence of osteonecrosis in the model and PNS group was 10/13 (76.92) and 4/15 (26.67%), respectively, indicating a significant difference between the two groups (*P* < 0.05).

### 3.3. Blood Biochemical Test Results

The biochemical indexes were illustrated in Tables 1 [Table tab2] [Table tab3] [Table tab4]–5. Compared with the controls, in the model group, the SOD activity, GSH-PX activity, T-AOC level, and inhibition of ·OH significantly decreased in a time-dependent manner, which then slightly rebounded on day 14, while the MDA content gradually increased and somewhat fell back on day 14 (*P* < 0.01). PNS could counterwork such changes, with a significant difference compared with the model group (*P* < 0.05, *P* < 0.01). These results indicated that, after steroid use, the animal body was in a state of oxidative stress, with stronger oxidation but weaker antioxidation, while PNS could inhibit oxidation and enhance antioxidation.

### 3.4. Biochemical Results of the Femoral Head

The oxidative and antioxidative indicators were shown in Tables 6 [Table tab7] [Table tab8] [Table tab9]–10. As was indicated, in the model group, after steroid injection, the SOD activity, GSH-PX activity, inhibition of ·OH, and T-AOC level gradually reduced in a time-dependent manner, while MDA content increased and remained at a high level. There were statistical differences between the control and model group (*P* < 0.05). In the PNS group, the abovementioned changes were downregulated, with significant difference between the model and PNS group (*P* < 0.05). These results suggested that the antioxidative activity of body was inhibited after steroid use and the body was in a state of oxidative stress, while PNS could mitigate the oxidation in the femoral head.

## 4. Discussion

In recent years, wide clinical use of steroid drugs has caused increasing incidence of SANFH. As one of the refractory diseases with high disability rate, SANFH has aroused great interest. Although orthopedists and researchers have actively investigated into the mechanism of SANFH and proposed many different theories [1–4], the exact pathogenesis of SANFH remains obscure, which has caused difficulties for the treatment and prevention of the disease.

Failure to expound the exact pathogenesis of SANFH is involved with the way models are established. Establishing desirable, standard, and repeatable models is essential to understand the pathogenesis of SANFH and select feasible plan of management. Since establishing models has problems of long period, high cost, high death rate, and low success rate, it is a long-term challenge for researchers, who have disputes on whether to combine steroid with equine serum [8, 9]. We supported the approach designed by Matsui et al. [10], which used equine serum twice as an auxiliary agent to trigger the pathological changes of the blood system and vascular system. Models established this way are in more accordance with the actual conditions of onset and treatment of SANFH and are consistent with the epidemiological features of SANFH. This approach has been accepted and adopted by the majority of researchers. It takes shorter time and produces higher success rate; however, the death rate of animals is increased [11]. Based on previous studies and experiences, we adopted a modified way for model establishment: two weeks after the second injection of 6 mL/kg equine serum, 45 mg/kg methylprednisolone was consecutively injected once a day for 3 days [7]. Histological and MRI results proved that this approach had high success rate, fewer complications, and lower death rate. Our way of establishing models is the key to the success of our experiment.

Oxidative stress is defined as an intracellular or extracellular state which can induce the generation of reactive oxygen species (ROS) [12]. In normal biological system, there is a balance between ROS production and detoxification by antioxidants, such as superoxide dismutase (SOD), catalase (CAT), glutathione peroxidase (GSH-PX), and glutathione. If this balance is broken, caused by excessive ROS, weakened detoxification, or dysfunction of antioxidant system, oxidative stress will occur, leading to instant or irreversible cell damage.

In normal life activities, the body constantly produces ROS. Meanwhile, its antioxidant defense system constantly detoxifies excessive ROS, maintaining a comparatively stable dynamic balance. When the balance is broken, oxidative stress will occur and oxidative damage will occur, causing functional disturbance and various diseases [13, 14]. Therefore, oxidative stress as a potential cause for SANFH should be considered.

Ichiseki et al. [15] found that, in the early stage of SANFH, GSH significantly decreased while LPO increased; besides, DNA oxidative damage was observed in local tissues. We held that there were blemish and disadvantage of this research: models induced by simple steroid use failed to simulate the clinical pathological process, and the indicators were partial and unable to observe the pathological state of oxidative stress dynamically from multidimensions.

Our research was designed to observe the dynamic changes of oxidation and antioxidation from both the holistic level and the local level in the femoral head. The first parameter was T-AOC level, which could effectively reveal the oxidative stress from the holistic and local levels. Our results indicated that, in the model group, the overall antioxidant ability decreased, and the antioxidant ability in the femoral head decreased as well. Therefore, we reckoned that steroid decreased the activities of antioxidative enzymes, and their ability to detoxify ROS was correspondingly weakened, which then caused oxidative stress in the whole body and the local bone tissues. These results have not been reported in previous studies, and more needs to be investigated.

Antioxidant enzymes are essential in the antioxidant defense system, which are able to detoxify ROS and prevent oxidative damage. Of the enzymes, SOD and GSH-PX play important roles.

Our results showed that, after steroid use, as time passed, both the holistic and the local levels of SOD and GSH-PX reduced significantly in the model group as compared with the control group, and there was statistical difference (*P* < 0.05). On day 14 after steroid use, the antioxidant indicators in the blood tended to recover, but those in the femoral head remained at a low level. We concluded that, after steroid use, the activities of antioxidant enzymes decreased, their ability to detoxify ROS was weakened, and thus the antioxidant ability reduced, making the whole body and local bone tissues in a state of oxidative stress. Although the overall antioxidative ability recovered somewhat finally, it remained low in the local bone tissues. What is worse, the bone tissues were unable to reverse the process, and then oxidative damage constantly existed and formed a vicious circle and in the end resulted in bone necrosis.

The above results indicated that steroid use caused weakened antioxidant ability in the whole body and local tissues. In order to observe whether steroid use could cause bidirectional change and produce excessive ROS, we chose MDA and ·OH as indicators of oxidation. MDA is one of the products present after oxygen free radicals attack polyunsaturated fatty acid in cell membrane. By detecting the content of MDA, we can know the extent of lipid overoxidation and intracellular damage [16, 17]. As one of the major causes for DNA oxidative damage [18], ·OH can directly react with nuclear DNA and change, damage, and exfoliate bases by hydrogen extraction and addition and electron flow. ·OH can damage mitochondrial DNA and affect the normal function of respiratory chain, causing deficiency of cellular energy and cell damage. Besides, ·OH can affect amino acid modification and degeneration; therefore, it is a potential source for protein damage.

Our results showed that, in the model group, both the holistic and the local levels of MDA were significantly elevated and the ability to inhibit ·OH was reduced. On day 14, MDA and ·OH levels in the blood tended to recover, while they remained at a high level in the femoral head. The results indicated that steroid use could generate excessive ROS, which could not be timely detoxified. As a result, the accumulated ROS broke the oxidation-reduction balance, and the femoral head was constantly damaged by lipid overoxidation, DNA cellular damage, and protein damage. In the end, necrosis occurred. We also speculated that, after steroid use caused oxidative stress and damage, some regulator genes might be initiated, or gene mutation might be triggered, which then led to SANFH.

As SANFH is a progressive disease, patients with advanced SANFH have to receive total hip arthroplasty (THA). The most fundamental and desirable approach of therapy is to prevent or stop bone necrosis with medications from the very origin and thus avoid or postpone THA. Referring to known pathogenesis, researches on preserving femoral head with Western medicines and preventing SANFH have achieved progress. However, long-term use of Western medicines needs to dynamically monitor, evaluate, and predict the risk factors. Besides, long-term use of Western medicines may cause certain toxic and side effects. What is worse, these medications impede the treatment of other concurrent diseases or even cause unexpected complications [19, 20]. To find better therapeutic approaches, some Chinese researchers turn their focus to Chinese medicines.

Only few researches have explored on antioxidant medications of SANFH. Ichiseki [21, 22] established rabbit SANFH models with single large-dose of steroid injection. When GSH was prophylactically supplemented, microcirculation injury within the femoral head was improved and incidence of SANFH was reduced. Kuribayashi et al. [23] held the fact that oxidative stress was involved in the pathogenesis of SANFH. In their research, they established SANFH models with single large-dose of steroid (20 mg/kg) and chose MDA only as an indicator. They observed that, 4 weeks after steroid use, MDA content in both serum and femoral head was elevated. Then they supplemented rabbit feeding stuff with Vitamin E as the antioxidant agent. After 6 weeks of prophylactic use, they found that MDA content was significantly reduced and incidence of bone necrosis was also reduced. However, there existed shortages in such researches: models induced by single use of steroid could not effectively imitate the clinical pathological process of SANFH, because the parameters only partially reflected oxidative stress from a certain perspective. Therefore, the results of such experiments are not convincing and we need to carefully select the parameters to observe the process of oxidation-antioxidation from multiple dimensions.

PNS is an active ingredient extracted from Chinese herbal medicine* Panax notoginseng*. It has advantages of stable structure, easy access, and low toxicity. It is widely used in clinical practice and is safe for short-term large-dose use or long-term use. So far researches have focused on the saponin content of* Panax notoginseng* [24]. In recent years, the antioxidant effect of PNS has drawn much attention and PNS has become a new herbal antioxidative medicine [25–27]. In our research, we chose to observe whether PNS had protective effect on SANFH.

Previous* in vitro* studies have shown that PNS could effectively detoxify ·OH and superoxide anions, meanwhile enhancing the activity of antioxidative enzymes [28]. Furthermore, PNS could inhibit the production of oxygen free radicals and improve the antioxidation ability of body, thus protecting cardiocerebrovascular system, liver, and kidneys [29, 30].

Our study found that prophylactic use of PNS could remarkably elevate the activities of SOD, GSH-PX, and general antioxidative ability in the whole body and femoral head. In the meantime, it could markedly improve the ability to inhibit ·OH and reduce MDA content. Our findings are consistent with the antioxidant effect of PNS reported in literature.

Our analysis is below. The major active ingredients of PNS are ginsenoside Rb1 (molecular formula: C_54_H_92_O_23_, molecular weight: 1109.31), ginsenoside Rg1 (molecular formula: C_42_H_72_O_14_, molecular weight: 801.02), and notoginsenoside R1 (molecular formula: C_47_H_80_O_18_, molecular weight: 933.14). Quite a few pharmacological studies have reported that these ingredients have strong antioxidant effects [31–33]. Previous comparative studies on PNS and the single ingredient also proved that PNS had stronger synergistic antioxidant effect than single ingredient. We proposed that when short-term large-dose of steroid was needed to treat certain disease, PNS should be used concurrently. In this way, the therapeutic effects of steroid drugs could be used, and their potential to cause SANFH could also be avoided.

Therefore, we concluded that, by boosting the activities of antioxidative enzymes and reducing the overgeneration of ROS, PNS could regulate the imbalance of oxidation-reduction and prevent further damage on necrotic bone tissues, thus playing a protective role on early SANFH. This mechanism will provide a novel theoretic and preventative strategy for SANFH.

The most fundamental and desirable approach of therapy is to prevent or stop bone necrosis with medications from the very origin in order to avoid or postpone THA. However, there exist limitations in researches on preserving femoral head with Western medicines and preventing SANFH. Therefore, some Chinese researchers have turned their focus to Chinese medicines for better therapeutic effects.

Certain progress has so far been achieved in the treatment and prevention of SANFH with Chinese medicine, but further* in vitro* and* in vivo* studies are still needed. We believe it is promising to investigate into this field. Faced with this tenacious enemy of human health, we should change the traditional habits of thinking and study SANFH from a holistic view. It is significant to explore the pathogenesis of SANFH from the molecular biological perspective combined with pharmacological studies on Chinese herbal medicines, so as to obtain the most desirable therapeutic effects and best prevent the occurrence of SANFH.

Our research has proved with animal experiments that PNS has protective effect on early SANFH by antioxidative pathway. However, much remains to be explored. We are confident that, with more in-depth pharmacological researches, PNS will be more widely used, effectively treating and preventing SANFH.

## Figures and Tables

**Table 1:** Serum T-AOC results of the three groups (u/mL).

Groups	0 d	3 d	5 d	7 d	14 d
Control	8.04 ± 0.71	8.17 ± 0.76	8.09 ± 0.75	8.21 ± 0.79	8.15 ± 0.69
Model	7.36 ± 0.47	6.32 ± 0.45^**^	6.01 ± 0.42^**^	5.81 ± 0.41^**^	7.03 ± 0.39^**^
PNS	7.34 ± 0.41	7.12 ± 0.44^∗#^	7.01 ± 0.48^∗##^	7.23 ± 0.45^∗##^	7.78 ± 0.51^#^

^*^Compared with the control group, *P* < 0.05; ^**^compared with the control group, *P* < 0.01; ^#^compared with the model group, *P* < 0.05; ^##^compared with the model group, *P* < 0.01.

**Table 2 tab2:** Serum SOD activity of the three groups (u/mL).

Groups	0 d	3 d	5 d	7 d	14 d
Control	250.36 ± 21.13	248.21 ± 19.37	252.36 ± 24.18	245.73 ± 20.74	247.04 ± 18.97
Model	235.12 ± 19.71	201.24 ± 14.78^**^	196.21 ± 15.73^**^	192.67 ± 14.21^**^	209.54 ± 17.12^**^
PNS	232.69 ± 21.61	220.41 ± 15.02^*^	215.19 ± 16.12^*^	219.34 ± 18.86^∗#^	231.17 ± 20.69^#^

^*^Compared with the control group, *P* < 0.05; ^**^compared with the control group, *P* < 0.01; ^#^compared with the model group, *P* < 0.05.

**Table 3 tab3:** Serum GSH-PX activity of the three groups (u/mL).

Groups	0 d	3 d	5 d	7 d	14 d
Control	331.04 ± 21.35	334.16 ± 23.92	332.87 ± 20.36	329.38 ± 21.69	332.29 ± 19.68
Model	320.29 ± 19.18	289.54 ± 16.75^**^	282.34 ± 16.15^**^	276.91 ± 15.17^**^	305.62 ± 16.89^*^
PNS	324.93 ± 20.47	295.23 ± 17.82^**^	296.28 ± 16.62^∗∗#^	298.21 ± 18.42^∗#^	318.19 ± 17.12^#^

^*^Compared with the control group, *P* < 0.05; ^**^compared with the control group, *P* < 0.01; ^#^compared with the model group, *P* < 0.05.

**Table 4 tab4:** Serum inhibition of ·OH of the three groups (u/mL).

Groups	0 d	3 d	5 d	7 d	14 d
Control	551.56 ± 41.54	540.58 ± 42.79	546.56 ± 45.51	551.68 ± 47.21	549.23 ± 39.19
Model	526.16 ± 35.63	441.23 ± 28.67^**^	436.23 ± 29.82^**^	424.43 ± 23.65^**^	478.52 ± 29.64^**^
PNS	521.92 ± 32.74	478.34 ± 35.15^∗∗#^	482.37 ± 28.31^∗∗#^	502.78 ± 26.97^∗##^	527.42 ± 31.58^#^

^*^Compared with the control group, *P* < 0.05; ^**^compared with the control group, *P* < 0.01; ^#^compared with the model group, *P* < 0.05; ^##^compared with the model group, *P* < 0.01.

**Table 5:** Serum MDA content in the three groups (nmol/mL).

Groups	0 d	3 d	5 d	7 d	14 d
Control	4.73 ± 0.67	4.51 ± 0.81	4.61 ± 0.71	4.77 ± 0.91	4.52 ± 0.75
Model	4.78 ± 0.63	7.36 ± 1.02^**^	7.87 ± 1.52^**^	8.93 ± 1.73^**^	6.85 ± 0.87^**^
PNS	4.80 ± 0.57	5.97 ± 0.96^∗#^	6.47 ± 1.42^∗#^	6.12 ± 0.89^∗##^	5.14 ± 0.89^##^

^*^Compared with the control group, *P* < 0.05; ^**^compared with the control group, *P* < 0.01; ^#^compared with the model group, *P* < 0.05; ^##^compared with the model group, *P* < 0.01.

**Table 6:** T-AOC results in the femoral head of the three groups (u/mg prot).

Groups	0 d	3 d	5 d	7 d	14 d
Control	1.68 ± 0.21	1.73 ± 0.32	1.69 ± 0.27	1.71 ± 0.19	1.70 ± 0.26
Model	1.63 ± 0.24	1.44 ± 0.18^*^	1.36 ± 0.18^*^	1.12 ± 0.15^**^	1.08 ± 0.19^**^
PNS	1.59 ± 0.25	1.51 ± 0.21^*^	1.43 ± 0.22^*^	1.34 ± 0.14^∗∗#^	1.50 ± 0.19^∗##^

^*^Compared with the control group, *P* < 0.05; ^**^compared with the control group, *P* < 0.01; ^#^compared with the model group, *P* < 0.05; ^##^compared with the model group, *P* < 0.01.

**Table 7 tab7:** SOD activity in the femoral heads of the three groups (u/mg prot).

Groups	0 d	3 d	5 d	7 d	14 d
Control	38.78 ± 3.25	40.23 ± 3.98	38.28 ± 3.95	39.91 ± 2.25	38.07 ± 3.39
Model	37.62 ± 2.19	34.27 ± 2.24^**^	32.89 ± 2.14^**^	31.69 ± 2.06^**^	32.15 ± 1.37^**^
PNS	36.97 ± 2.78	35.69 ± 2.67^*^	34.54 ± 2.25^*^	36.29 ± 1.98^∗##^	35.97 ± 2.06^∗##^

^*^Compared with the control group, *P* < 0.05; ^**^compared with the control group, *P* < 0.01; ^#^compared with the model group, *P* < 0.05; ^##^compared with the model group, *P* < 0.01.

**Table 8 tab8:** GSH-PX activity in the femoral heads of the three groups (u/mg prot).

Groups	0 d	3 d	5 d	7 d	14 d
Control	107.24 ± 8.74	112.42 ± 10.76	109.24 ± 8.46	109.96 ± 10.06	110.27 ± 9.96
Model	101.53 ± 9.78	86.76 ± 4.26^**^	84.26 ± 4.06^**^	79.09 ± 4.71^**^	60.35 ± 3.74^**^
PNS	98.37 ± 10.21	87.24 ± 5.97^*^	89.01 ± 6.02^∗#^	95.34 ± 6.86^∗##^	94.57 ± 7.01^##^

^*^Compared with the control group, *P* < 0.05; ^**^compared with the control group, *P* < 0.01; ^#^compared with the model group, *P* < 0.05; ^##^compared with the model group, *P* < 0.01.

**Table 9 tab9:** Inhibition of ·OH in the femoral heads of the three groups (u/mg prot).

Groups	0 d	3 d	5 d	7 d	14 d
Control	64.27 ± 8.71	65.31 ± 9.95	64.34 ± 8.91	62.78 ± 9.32	65.19 ± 9.07
Model	61.65 ± 9.28	47.43 ± 5.14^**^	43.16 ± 4.36^**^	40.29 ± 5.21^**^	41.08 ± 4.79^**^
PNS	59.78 ± 10.21	50.89 ± 5.62^**^	49.43 ± 4.75^∗∗#^	54.96 ± 5.01^∗##^	53.61 ± 6.23^∗##^

^*^Compared with the control group, *P* < 0.05; ^**^compared with the control group, *P* < 0.01; ^#^compared with the model group, *P* < 0.05; ^##^compared with the model group, *P* < 0.01.

**Table 10:** MDA Contents in the femoral heads of the three groups (nmol/mg prot).

Groups	0 d	3 d	5 d	7 d	14 d
Control	9.94 ± 0.71	10.05 ± 0.76	9.79 ± 0.83	10.25 ± 0.73	10.12 ± 0.89
Model	10.98 ± 0.67	12.87 ± 0.98^**^	12.76 ± 0.89^**^	13.42 ± 0.76^**^	13.16 ± 1.24^**^
PNS	10.12 ± 0.43	11.32 ± 0.74^∗#^	11.67 ± 0.71^∗#^	11.09 ± 0.69^∗##^	11.23 ± 0.87^∗#^

^*^Compared with the control group, *P* < 0.05; ^**^compared with the control group, *P* < 0.01; ^#^compared with the model group, *P* < 0.05; ^##^compared with the model group, *P* < 0.01.
